# Highly Efficient Catalytic Oxidation of Glucose to Formic Acid over Mn-Mo Doped Carbon Nanotube

**DOI:** 10.3390/molecules30071639

**Published:** 2025-04-07

**Authors:** Hongrui Guo, Fan Yang, Siwei Chen, Hejuan Wu, Jirui Yang, Feng Shen

**Affiliations:** 1 Agro-Environmental Protection Institute, Chinese Academy of Agricultural Sciences, No. 31 Fukang Road, Nankai District, Tianjin 300191, China; g1620344175@163.com (H.G.); siweichen0115@163.com (S.C.); 2 College of Resources and Environment, Huazhong Agricultural University, Wuhan 430070, China; 18342852183@163.com; 3 School of Energy and Environmental Engineering, Hebei University of Technology, Tianjin 300401, China; wuhejuan1112@163.com

**Keywords:** lignocellulose, carbohydrates, manganese oxides, bimetallic oxides

## Abstract

The production of formic acid (FA) from lignocellulose and its derived sugars represents a pivotal upgrading reaction in biorefinery. This work prepared a Mn-Mo doped carbon nanotube composite catalyst for the catalytic oxidation of glucose into FA in an O_2_ atmosphere, under extremely low Mn (3.27%) and Mo (0.40%) loading conditions, displaying a comparable performance with the traditional vanadium-based catalyst suffering from toxicity issues. It was confirmed that the doping of Mo led to the formation of MnMoO_X_ and increased the contents of low-valence Mn species (Mn^2+^ + Mn^3+^), lattice oxygen (O_latt_), and surface adsorbed oxygen (O_ads_) based on various characterization methods, such as XRD, XPS, TEM and ICP, which were beneficial to improve the catalytic performance. The maximum FA yield of 58.8% could be achieved over Mn_9_Mo_1_O_X_@MWCNT after reaction for 6 h at 140 °C, which was far more than that obtained with undoped MnO_X_@MWCNT (14.5%) at the identical conditions. Glyoxylic acid and arabinose were identified as two main intermediates, suggesting that the transformation of glucose into FA over Mn_9_Mo_1_O_X_@MWCNT involved two different paths. This work proved that manganese-based catalyst was a green alternative for upgrading lignocellulose via catalytic oxidation.

## 1. Introduction

To address the energy crisis and mitigate greenhouse gas emissions, lignocellulosic biomass, as an abundant and renewable resource, has garnered significant attention for its potential to produce a range of high-value chemicals and fuels [1,2]. Among the components of lignocellulosic biomass, polysaccharides such as cellulose and hemicellulose, along with their derivatives, have been extensively utilized to produce versatile chemicals, including levulinic acid [3,4], furan [5], 5-HMF [6], ethanol [7,8], lactic acid [9], and formic acid [10,11]. Amongst these, formic acid (FA) is widely used in hydrogen energy storage [12,13], fuel cells [14], textile, leather, medicine, agriculture and rubber [15]. FA can be further decomposed into CO or H_2_, with the former widely used in the semiconductor industry and H_2_ being an important energy carrier [16,17]. Currently, the industrial production of FA is mainly carried out through the methyl formate hydrolysis method [18] with its primary raw materials being derived from fossil fuels. In contrast, the conversion of lignocellulose into FA represents a sustainable and environmentally friendly alternative [10,19,20].

The catalytic oxidation of lignocellulose and its derived sugars to FA has been most widely carried out over vanadium-based catalysts in the presence of O_2_, such as heteropoly acids [21,22], NaVO_3_ [23], VOSO_4_ [24]; however, the biological toxicity and corrosiveness of vanadium are still limiting issues. In addition, a number of catalysts based on metal oxides, including MgO [25], CaO [25], and CuO_X_/MgO [26], have been excavated. These processes generally use a high concentration of H_2_O_2_ as oxidants, and the production of OH and $O_{2}^{-}$ depends on the presence of NaOH [27,28,29]. Therefore, it needs a high concentration of alkali as an accelerator in this system, suffering from a big challenge of high alkali pollution [30]. Therefore, it is necessary to develop a greener and more efficient catalytic system, especially utilizing O_2_ as the oxidant.

Manganese oxides (MnO_X_) as a kind of transition metal oxides have been proven to show high stability, great activity, low-cost, and green catalysts [31] in the application of gas pollutant decomposition [32,33], heavy metal adsorption [34], and organic pollutant degradation [35], which is attributed to their different morphologies, surface elemental compositions, specific surface area, and oxygen vacancy [36]. To further increase the catalytic performance, a secondary metal such as Mo, Ce, Fe, or Cu is deliberately incorporated, which can improve both redox and acid properties [37]. Guo et al. [38] reported the preparation of Mo-modified MnO_X_ catalyst and confirmed that Mo increased the proportion of low-valence Mn (Mn^2+^ + Mn^3+^) and O_ads_, thereby enhancing the catalytic activity, with a FA yield of 54% compared to 33% over the MnO_X_ catalyst. Additionally, Mn_4_Ce_0.05_O_X_ prepared by Xu et al. has also been observed to increase the yield of FA from 40.5% to 65.1% [39]. The application of suitable carriers is a prevalent strategy to enhance the dispersion and activity of metals/metal oxides [40,41]. The carbonaceous supports, such as carbon fibers, carbon nanotubes, and graphene, have been widely used to disperse MnO_X_ nanoparticles, due to their attractive properties of uniform surface morphology and high conductivity [42,43,44]. Introducing metals onto multi-walled carbon nanotubes (MWCNT) can significantly improve catalytic activity by increasing the number of active sites available for reactions [45,46].

In this work, we synthesized a Mo-doped MnO_X_ composite supported on a multi-walled carbon nanotube (MnMoO_X_@MWCNT), which was utilized in the catalytic oxidation of glucose into FA in the presence of O_2_. The maximum FA yield was 58.8% for 6 h at 140 °C under extremely low Mn (3.27%) and Mo (0.40%) loading conditions and was comparable to the yields of vanadium-based catalysts. The key role of doped Mo in the improvement was demonstrated, and the catalytic conversion process was discussed in detail. This work would provide an efficient and sustainable method for the upgrading of lignocellulose and its derivatives into FA.

## 2. Results

### 2.1. Characterizations of Catalysts

**Scheme 1:**
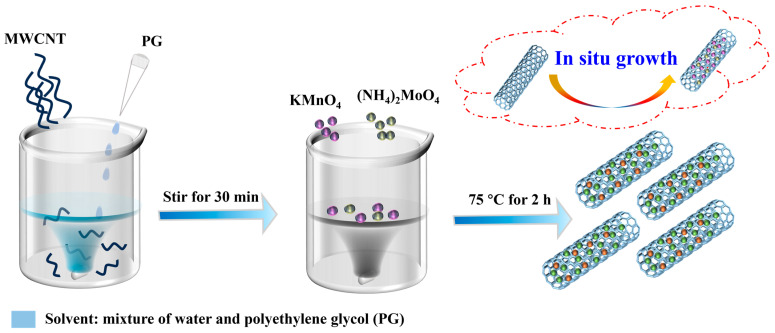
Schematic illustration of the synthesis of MnMoO_X_@MWCNT.

**Figure 1:**
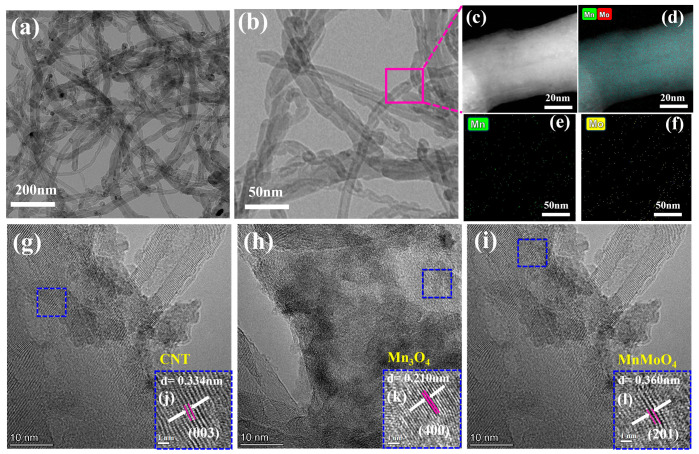
TEM images of (**a**) MWCNT, (**b**) Mn_9_Mo_1_O_X_@MWCNT; (**c**) HAADF-STEM image with (**d**–**f**) EDS analysis of Mn_9_Mo_1_O_X_@MWCNT; (**g**–**i**) TEM images of Mn_9_Mo_1_O_X_@MWCNT; HRTEM images of (**j**) MWCNT, (**k**) Mn_3_O_4_, (**l**) MnMoO_4_.

The Mn_(a)_Mo_(b)_O_X_@MWCNT was synthesized via a method that facilitated the growth of MnMoO_X_ on MWCNT substrates, as illustrated in Scheme 1 [46]. TEM images (Figure 1a,b) showed the morphological structure of the original MWCNT and the Mn_9_Mo_1_O_X_@MWCNT catalyst. For Mn_9_Mo_1_O_X_@MWCNT, metal particles were not visible attributing to lower Mn (3.27%) and Mo (0.40%) loading. The mapping analysis of Mn_9_Mo_1_O_X_@MWCNT (Figure 1d–f) confirmed that both Mn and Mo were evenly distributed on the MWCNT. Further, the HRTEM image disclosed an interlayer spacing of 0.334 nm for Mn_9_Mo_1_O_X_@MWCNT, corresponding to the (003) plane of the MWCNT (Figure 1j) [47]. The lattice fringes of 0.210 nm and 0.360 nm corresponded to the (400) plane of the Mn_3_O_4_ [48] and the (201) plane of the MnMoO_4_ (Figure 1k,l) [49], respectively, indicating the formation of mixed oxides.

**Figure 2:**
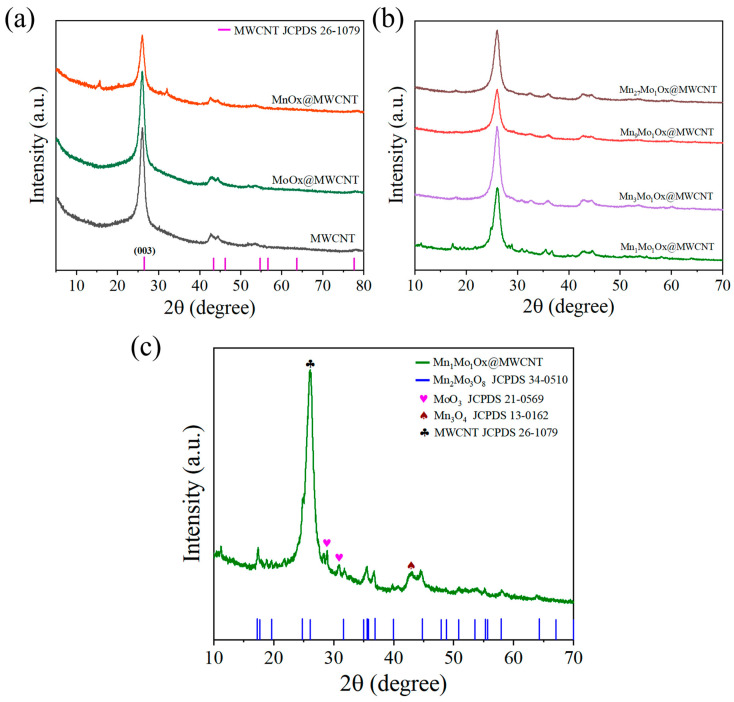
XRD patterns of (**a**) MWCNT and single metal load, (**b**) various Mn_(a)_Mo_(b)_O_X_@MWCNT samples, and (**c**) Mn_1_M_1_O_X_@MWCNT.

The XRD patterns showed that the sharp peaks at 2θ = 26.60°, 43.45° and 46.33°, corresponding to diffraction from the (003), (101) and (012) planes of CNT (JCPDS card, No. 26-1079) [47], appeared in all samples (Figure 2a). For MnO_X_@MWCNT and MoO_X_@MWCNT, no significant differences were observed in the XRD pattern, likely attributed to the good dispersion of metals on the MWCNTs [50]. By contrast, some new peaks were observed in Mn_(a)_Mo_(b)_O_X_@MWCNT, and their intensities were increased with the decreasing Mn/Mo ratio (Figure 2b). Especially for Mn_1_Mo_1_O_X_@MWCNT, the additional peaks at 17.3°, 31.6°, 35.6°, 36.8° and 44.8° corresponded to the (002), (103), (112), (201) and (203) crystal planes of the hexagonal Mn_2_Mo_3_O_8_ (JCPDS card, No.34-0510) [51] (Figure 2c). The peaks at 29.4° and 31.0° were attributed to (300) and (204) crystal faces of MoO_3_ (JCPDS card, No. 21-0569) [52] along with a fraction of the Mn_3_O_4_ phase (JCPDS card, No. 13-0162) [53]. The actual ratio of Mn to Mo was also determined by ICP MS and the results showed that theoretical value and the results were consistent except for Mn_27_Mo_1_O_X_@MWCNT (Table 1).

**Figure 3:**
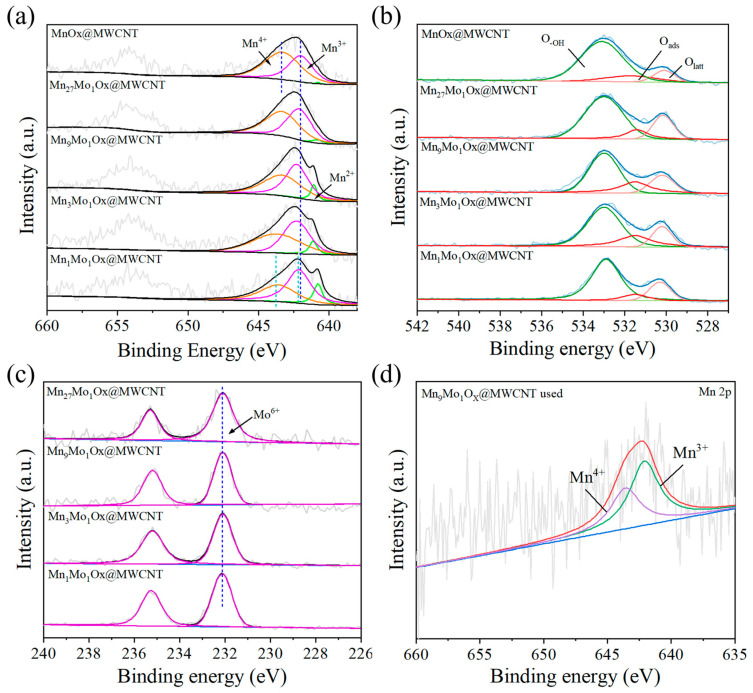
XPS spectra of prepared samples: (**a**) Mn 2p, (**b**) O 1s, (**c**) Mo 3d, (**d**) Mn 2p of Mn_9_Mo_1_O_X_@MWCNT after the third cycle.

XPS analysis was performed to investigate the different valence states of surface Mn, Mo, and O in a series of catalysts (Figure 3). The spectrum of Mn 2p (Figure 3a) showed three distinct peaks centered at around 640.8 eV, 642.4 eV, and 643.8 eV, corresponding to Mn^2+^, Mn^3+^, and Mn^4+^ [54], and the proportion of these species was summarized in Table 2. The XPS spectrogram of Mo 3d was shown in Figure 3c, the peaks at 232.2 eV and 235.2 eV were attributed to Mo^6+^ [55], and these two peaks did not shift significantly. MnO_X_@MWCNT contained approximately 41.3% low-valence Mn (3.1% Mn^2+^ + 37.6% Mn^3+^) and 58.7% Mn^4+^. With the addition of Mo, Mn 2p peaks of Mn_(a)_Mo_(b)_O_X_@MWCNT shifted toward higher binding energy compared with MnO_X_@MWCNT, suggesting the formation of a strong interaction between Mn and Mo [56]. Meanwhile, the ratio of (Mn^2+^ + Mn^3+^)/Mn trended upwards in accordance with the increase in Mo content (41.3–64.3%), which was found to be highly related to the performance of the catalyst [39,57].

Figure 3c demonstrated the O 1s peaks at approximately 530.1 eV, 531.4 eV, and 532.5 eV, which were assigned to lattice oxygen (O_latt_), surface adsorbed oxygen (O_ads_), and oxygen in water molecules (O_−OH_) [58,59]. Both O_latt_ and O_ads_ were involved in the catalytic oxidation and the mutual conversion between them under certain conditions played a major role in the catalytic oxidation process [60,61]. As shown in Table 2, the incorporation of Mn alone resulted in a content of 8.8% O_latt_ and 5.2% O_ads_. However, following the addition of Mo, there was a substantial increase in the content of O_latt_ and O_ads_, with Mn_9_Mo_1_O_X_@MWCNT exhibiting the highest concentrations of O_latt_ (20.2%) and O_ads_ (23.6%). This observation was particularly advantageous for subsequent catalytic oxidation reactions [62]. In summary, doping with the appropriate amount of Mo was beneficial to increase the content of O_latt_ and O_ads_ in the catalyst, and caused a variation in the metal valence on the surface, thus adjusting the redox properties [63].

### 2.2. Catalytic Oxidation of Glucose to FA

**Figure 4:**
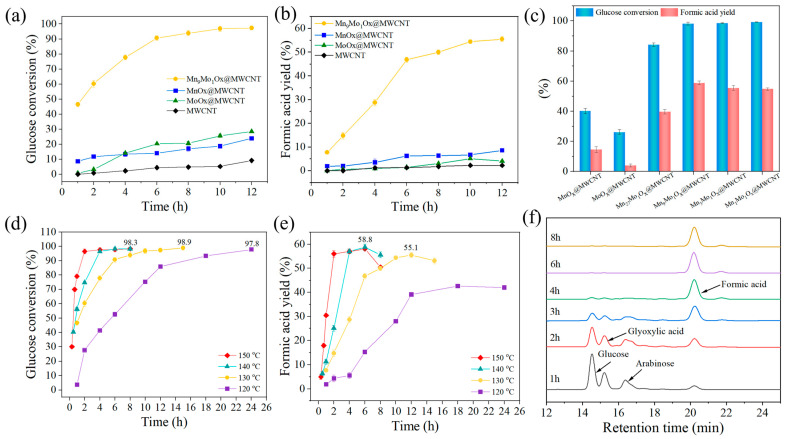
Catalytic activity of various Mn_(a)_Mo_(b)_O_X_@MWCNT samples (**a**) glucose conversion, (**b**) FA yield (conditions: 100 mg glucose, 50 mg catalyst, 5 mL H_2_O, 130 °C, 3 MPa O_2_); (**c**) catalytic activity of different catalysts under the same conditions (conditions: 100 mg glucose, 50 mg catalyst, 5 mL H_2_O, 140 °C, 6 h, 3 MPa O_2_); catalytic activity of Mn_9_Mo_1_O_X_@MWCNT in different temperature; (**d**) glucose conversion; (**e**) FA yield (conditions: 100 mg glucose, 50 mg catalyst, 5 mL H_2_O, 3 MPa O_2_); (**f**) HPLC spectrum of Mn_9_Mo_1_O_X_@MWCNT in 140 °C.

Using glucose as a substrate, the catalytic oxidation was tested for 12 h at 130 °C (Figure 4a). When MWCNT was utilized as a catalyst, a gradual increase in glucose conversion happened and reached approximately 10% with no yield of FA. For MnO_X_@MWCNT and MoO_X_@MWCNT, the glucose conversion could ultimately be increased to 20–25%, but the FA yield remained less than 10%. In contrast, when Mn and Mo were added simultaneously at a ratio of 9:1, there was a marked enhancement in both glucose conversion (97.4%) and FA yield (55.5%) over Mn_9_Mo_1_O_X_@MWCNT.

The activity of catalysts with different Mo contents was determined, and the results are shown in Figure 4c. At a Mn/Mo ratio of 27, the glucose conversion could be increased to 84.2% and the FA yield was up to 39.7% over Mn_27_Mo_1_O_X_@MWCNT. When the Mn/Mo ratio was increased to 9, glucose was almost completely converted with 58.8% FA yield over Mn_9_Mo_1_O_X_@MWCNT. Further increase in the Mn/Mo ratio resulted in a decrease in the FA yield to 55.5% and 54.8%, respectively, for Mn_3_Mo_1_O_X_@MWCNT and Mn_1_Mo_1_O_X_@MWCNT. This may be due to the generation of MoO_3_, leading to a decrease in catalytic activity [39]. Based on the results of Figure 4c and Table 2, it was found that the low-valence Mn as well as O_latt_ and O_ads_ content were correlated with FA yield, which was consistent with our previous works [57]. The Mn_9_Mo_1_O_X_@MWCNT exhibited moderate low-valence Mn (54.2%) and the most O_latt_ and O_ads_ of 20.2% and 23.6%, showing the best activity among the prepared catalysts. In a word, the incorporation of an appropriate amount of Mo increased the ratio of (Mn^2+^ + Mn^3+^)/Mn, O_latt_, and O_ads_ in the catalyst, and adjusted the electronic structure, thereby improving the activity. Therefore, the appropriate Mn/Mo molar ratio (9/1) achieved the best catalytic effect under the given conditions, and subsequent experiments about the effects of reaction temperature and atmosphere conditions were conducted using this catalyst as an example.

**Figure 5:**
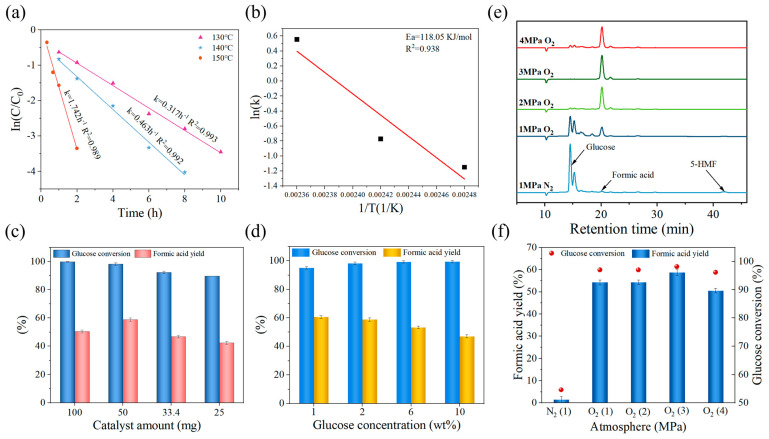
(**a**) First-order kinetic fit and (**b**) reaction activation energy for catalytic oxidation reaction of Mn_9_Mo_1_O_X_@MWCNT, the black square for reaction rate constant and red line for fit data; catalytic oxidation of (**c**) different catalyst amounts (conditions: 100 mg glucose, 5 mL H_2_O, 3 MPa O_2_, 140 °C, 6 h); (**d**) different concentration of glucose (conditions: 50 mg catalyst, 5 mL H_2_O, 3 MPa O_2_, 140 °C, 6 h); (**e**) HPLC spectrum of Mn_9_Mo_1_O_X_@MWCNT in different atmosphere; (**f**) different atmosphere (conditions: 100 mg glucose, 50 mg catalyst, 5 mL H_2_O, 140 °C, 6 h).

The catalytic oxidation of glucose over Mn_9_Mo_1_O_X_@MWCNT was conducted at the temperature of 120–150 °C (Figure 4d,e). At 120 °C, the conversion and FA yield were increased gradually with time and achieved a maximum FA yield of 42.6% within 18 h. After 12 h of reaction at 130 °C, the glucose conversion was 97.4%, and the FA yield was 55.1%. When the reaction temperature was raised to 140 °C and 150 °C, the highest yields of FA were 58.8% (140 °C) and 58.1% (150 °C) after 6 h, respectively. The preceding results demonstrated that elevated temperatures could expedite the reaction rate. However, elevated temperatures or extended duration may diminish the selectivity of FA due to the potential decomposition of FA or the generation of some unwanted substances within the system [22]. As indicated by the kinetics model fitting (Figure 5a,b), it was observed that elevating the reaction temperature from 130 °C to 150 °C resulted in a substantial increase in the reaction rate from 0.317 h^−1^ to 1.742 h^−1^. It could be demonstrated that an increase in reaction temperature can enhance the reaction rate while reducing the occurrence of side reactions, thus resulting in an enhancement of the yield [64]. The reaction activation energy was 118.05 KJ/mol, which was better than the energy needed for the Mn-Mo oxides (172.3 KJ/mol) [65]. Figure 5c shows the influence of the catalyst amount on its performance. The FA yield was increased with the increase in catalyst amount and reached the maximum value of 58.8% when 50 mg catalyst was loaded. Then, FA yield was decreased when the amount was further increased to 100 mg. Moreover, when the concentration of glucose reached 10 wt%, 47.8% yield of FA was obtained at 140 °C after reaction for 6 h, suggesting that the catalyst still had good tolerance to high glucose concentration (Figure 5d).

The atmosphere conditions were found to be pivotal in the catalytic oxidation of glucose (Figure 5f). After reaction for 6 h at 140 °C, glucose was hardly converted (<10%) over Mn_9_Mo_1_O_X_@MWCNT when 1 MPa of N_2_ was filled, and negligible FA (1.5%) was obtained. In contrast, at the same conditions except for the use of O_2_ atmosphere (1 MPa), 97.1% glucose was conversed and the FA yield was 54.3%. The FA yield was increased with the increasing of O_2_ pressure (1 to 3 MPa) with the maximum FA yield of 58.8% at 3 MPa, and then it was decreased when the O_2_ pressure was further increased to 4 MPa. Generally, the appropriate O_2_ pressure was beneficial to the oxidation process of the catalyst from the reduced state to the oxidized one, which was advantageous for the continuous generation of FA [20]. The products of glucose conversion under different atmosphere conditions were illustrated in the HPLC spectra (Figure 5e). It was found that when 1 MPa O_2_ was added, the products consisted of residual glucose, glyoxylic acid, and FA. With the O_2_ pressure was 2 to 4 MPa, the product was only FA, which indicated that the O_2_ atmosphere was helpful to the rapid conversion of glucose to FA, and glyoxylic acid might be one of the intermediate products, which will be further discussed later. When only N_2_ existed, a small amount of 5-HMF was detected in the product, indicating that glucose dehydration occurred. The gaseous products were collected for determination after the reaction at 140 °C for 6h with 3 MPa O_2_. Only small amounts of CO_2_ and CO were detected, corresponding to approximately 5% carbon balance in total. In addition, about 58.8% FA and 4.1% acetic acid were detected in the liquid. Therefore, approximately 67.9% carbon balance was finally obtained by a combination of both gaseous and liquid products. Several unknown products such as humic acid might be present in the system; however, it could not be recognized by our detection method [66,67].

The catalytic oxidation of other carbohydrates over Mn_9_Mo_1_O_X_@MWCNT was conducted, including monosaccharides (fructose, xylose, arabinose), disaccharides (cellobiose), polysaccharides (xylan) and microcrystalline cellulose (Table 3). Generally, using monosaccharides as the substrates all obtained satisfactory FA yields. After the reaction at 140 °C for 6 h, the FA yields of 49.9%, 57.3%, and 56.8% were obtained from the oxidation of fructose, xylose, and arabinose, respectively. For cellobiose, 34.4% FA was obtained at 140 °C for 6 h. When the temperature was raised to 160 °C, the FA yield could reach 52.3% within 4 h. In addition, xylan can also produce 50.7% FA at 140 °C for 6 h. Using microcrystalline cellulose pre-treated with ball milling and acid as the substrate, Mn_9_Mo_1_O_X_@MWCNT exhibited much lower catalytic performance with FA yield of 20.5% at 170 °C for 6 h and 23.5% at 180 °C for 4 h. A comparison with the reported literature (Table 4) revealed that Mn_9_Mo_1_O_X_@MWCNT can achieve considerable FA yields from the oxidation of glucose equivalent to traditional vanadium-based catalysts.

The recycling experiment for Mn_9_Mo_1_O_X_@MWCNT was tested, and the reused catalyst was characterized by XPS and ICP-MS. The results showed that after the third cycle, the glucose conversion and FA yield decreased to 60.7% from 91.8% and 21.5% from 46.8%, respectively. The XPS result (Figure 3d) showed that the signal of Mn became much weaker compared with that in the fresh catalyst (Figure 3a). ICP-MS analysis revealed that approximately 85% of Mn and 72% of Mo were lost, indicating the loss of metals easily happened in recycling. Thus, the decrease in catalytic performance could be attributed to the leaching of active metals on the surface of the catalyst. How to improve the stability of active metals is an urgent need to resolve in the future (Table 5).

### 2.3. Possible Pathways of Glucose Oxidation to FA over Mn_9_Mo_1_O_X_@MWCNT

**Figure 6:**
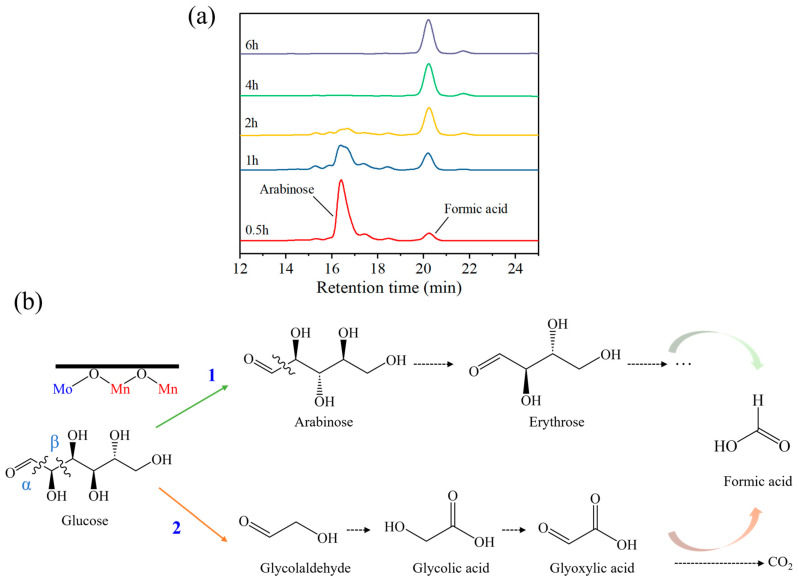
(**a**) UPLC spectra of oxidation of arabinose to FA (conditions: 100 mg arabinose, 50 mg catalyst, 5 mL H_2_O, 140 °C, 3 MPa O_2_); (**b**) possible reaction paths in water of glucose to FA by Mn_(a)_Mo_(b)_O_X_@MWCNT.

In order to explore the possible pathways of glucose oxidation to FA over Mn_9_Mo_1_O_X_@MWCNT, the HPLC spectrogram (Figure 4f) of different reaction times at 140 °C was analyzed. When the reaction time was 1 h, with the exception of two peaks at 14.5 min (glucose) and 20.2 min (FA), two obvious peaks of intermediates were observed, which gradually diminished over 6 h. By comparing with the standard substances, the two main intermediate products were identified as glyoxylic acid (15.2 min) and arabinose (16.5 min). Then, arabinose was used as the substrate for the catalytic oxidation reaction under identical conditions. However, the peak of glyoxylic acid (15.2 min) was not observed during the whole reaction time (Figure 6a), suggesting that the production of glyoxylic acid and arabinose proceeded in two different paths (Figure 6b). In path 1, the carbon bond at the α site was broken resulting in the decomposition of glucose into FA and the intermediate product arabinose. Subsequently, the carbon bonds of arabinose gradually broke down with the same pattern above, decomposing into FA and other by-products [72]. In pathway 2, glucose first underwent a continuous reverse aldol condensation reaction to produce ethanol, which was then converted into glycolic acid [73,74]. Finally, glycolic acid was changed into glyoxylic acid, which then turned into FA and CO_2_.

## 3. Materials and Methods

### 3.1. Materials

The carboxylated multi-walled carbon nanotubes were purchased from Chengdu Organic Chemicals Co. Ltd. (Chengdu, China). D(+)-Glucose (98%) from Aladdin Biochemical Technology Co., Ltd. (Shanghai, China). D-Fructose (99%), D(+)-Xylose (99%), ammonium molybdate ((NH_4_)_2_MoO_4_) (AR) from Macklin. Co., Ltd. (Shanghai, China). L(+)-Arabinose (98%) was purchased from Yuanye Bio-Technology Co., Ltd. (Shanghai, China). Potassium permanganate was obtained from Tianjin Kemiou Chemical Reagent Co., Ltd. (Tianjin, China). The water used in the experiment is ultra-pure water.

### 3.2. Synthesis of MnMoO_X_@MWCNT

The composite catalyst was prepared with a modified reduction method illustrated in Scheme 1 [46]. Typically, 10 mL polyethylene glycol (PG) and water with a volume ratio of 1:1 were added into a beaker and mixed evenly, and then, 500 mg of MWCNT was added, keeping stirring for 30 min (solution 1). Then, 55 mg KMnO_4_ and 8 mg (NH_4_)_2_MoO_4_ (Mn/Mo ratio was 9/1) were added into solution 1 successively and stirred at 75 °C for 2 h. Finally, the mixture cooled down to room temperature. The product was collected and washed with water and ethanol three times and vacuum-dried for 12 h at 80 °C. By changing Mo content, the Mn_(a)_Mo_(b)_O_X_@MWCNT were prepared, in which a/b = 1/1, 3/1, 9/1 and 27/1. Similar to the method above, MnO_X_@MWCNT and MoO_X_@MWCNT were prepared by adding only KMnO_4_ and (NH_4_)_2_MoO_4_, respectively.

### 3.3. Catalyst Characterization

Transmission electron microscopy (TEM) and mapping analysis were performed using the JEOL JEM 2100 brand device (Akishima City, Tokyo, Japan) to elucidate the nanocomposites’ morphological structure. Samples were deposited on Cu with C coating TEM grids, after grinding and suspending in ethanol. The X-ray diffraction (XRD, Bruker D8 Advance, Billerica, MA, USA) of the resulting product was characterized using Cu-Kα radiation (λ = 1.54056 Å) with an operating voltage of 40 kV and the working current of 40 mA during 5–90° at a scanning rate of 2°/min (Waltzbach, Berlin, Germany). X-ray photoelectron spectroscopy (XPS) analysis was conducted utilizing the Thermo Scientific ESCA-LAB 250Xi spectrometer (Thermo Fisher Scientific, Waltham, MA, USA), equipped with monochromatic Al Kα radiation. The content of different metal elements in the samples was determined using an Agilent 7800 inductively coupled plasma mass spectrometer (ICP-MS, Santa Clara, CA, USA). The contents of CO and CO_2_ were measured by Trace 1300 gas chromatograph (Thermo Fisher Scientific, Waltham, MA, USA), using an auxiliary valve box for process injection.

### 3.4. Catalytic Reaction and Product Analyses

All catalytic oxidation was conducted in the high-pressure reactors (SLF-35-260, Guizhou Shanli Instrument Company, Guizhou, China). Each high-pressure reactor contains a Teflon-lined vessel (35 mL), with adjustable reaction temperature (upper lower limit of 280 °C) and stirring speed (0–1500 rpm). In a typical process, 100 mg glucose, 50 mg catalyst, and 5 mL H_2_O were added into the autoclave. The air in the reactor was replaced with O_2_ several times and then maintained at 1–4 MPa, then heated at 130–160 °C while stirring. After the reaction, the reactor was placed in flowing cold water for cooling. The liquid product was filtered with a 0.22 μm filter membrane, and analyzed by ultra-performance liquid chromatography (Water Acquity UPLC, Milford, MA, USA), and the instrument was equipped with a column of Shodex SH1011 (Tokyo, Japan) and a refractive index detector. The temperatures of the column and RI detector were 50 °C and 35 °C. The mobile phase consisting of H_2_SO_4_ (5 mM) was maintained at a flow rate of 0.5 mL min^−1^. The conversion of glucose, the yield of FA, and the yield of AA were obtained with Equations (1)–(3):

(1) $$\mathrm{Glucose}\;\mathrm{conversion}\;(\%)=\frac{\mathrm{moles}\;\mathrm{of}\;\mathrm{reacted}\;\mathrm{glucose}}{\mathrm{initial}\;\mathrm{moles}\;\mathrm{of}\;\mathrm{glucose}}\;\times \;100\%$$

(2) $$\mathrm{Formic}\;\mathrm{acid}\;\mathrm{yield}\;(\%)=\frac{\mathrm{moles}\;\mathrm{of}\;\mathrm{FA}\;\mathrm{produced}}{\;\mathrm{initial}\;\mathrm{moles}\;\mathrm{of}\;\mathrm{substrates}\;\times \;6}\times \;100\%$$

(3) $$\mathrm{Acetic}\;\mathrm{acid}\;\mathrm{yield}\;(\%)=\frac{\mathrm{moles}\;\mathrm{of}\;\mathrm{AA}\;\mathrm{produced}}{\;\mathrm{initial}\;\mathrm{moles}\;\mathrm{of}\;\mathrm{substrates}\;\times \;3}\times \;100\%$$

## 4. Conclusions

This work prepared a Mn-Mo doped carbon nanotube composite catalyst (MnMoO_X_@MWCNT), which was highly efficient for the oxidation of glucose into FA with the optimal FA yield of 58.8% after reaction for 6 h at 140 °C. The maximum FA yield was 58.8% for 6 h at 140 °C under extremely low Mn (3.27%) and Mo (0.40%) loading conditions, displaying a compared catalytic performance with the traditional vanadium-based catalysts. The doping of Mo played a key role in improving the performance of MnMoO_X_@MWCNT by increasing the low-valence Mn (Mn^2+^ and Mn^3+^), O_ads_, and O_latt_ content. Transformation of glucose into FA over Mn_9_Mo_1_O_X_@MWCNT involved two different paths with glyoxylic acid and arabinose as the intermediates, respectively. Mn_9_Mo_1_O_X_@MWCNT also had good applicability for catalyzing the oxidation of various carbohydrates to FA. This work provided ideas for the design of Mn-based catalysts for the oxidation of lignocellulose and its derived sugars to FA in the future.

## Figures and Tables

**Table 1:** The molar ratio of Mn to Mo in different catalysts.

Catalyst	Ratio of Mn/Mo	
	Theoretical Value	Actual Value *^a^*
Mn_1_Mo_1_O_X_@MWCNT	1.0	1.0
Mn_3_Mo_1_O_X_@MWCNT	3.0	2.8
Mn_9_Mo_1_O_X_@MWCNT	9.0	8.2
Mn_27_Mo_1_O_X_@MWCNT	27.0	20.4

*^a^* Actual values were calculated based on ICP MS data.

**Table 2:** The composition of O and Mn elements on the surface of the prepared catalysts.

Catalyst	Mn		O	
	(Mn^2+^ + Mn^3+^)/Mn_tot_ (%)	Mn^4+^/Mn_tot_ (%)	O_ads_/O_tot_ (%)	O_latt_/O_tot_ (%)
MnO_X_@MWCNT	41.3	58.7	5.2	8.8
Mn_27_Mo_1_O_X_@MWCNT	47.9	52.1	13.3	19.3
Mn_9_Mo_1_O_X_@MWCNT	54.2	45.8	23.6	19.2
Mn_3_Mo_1_O_X_@MWCNT	56.2	43.8	22.8	16.3
Mn_1_Mo_1_O_X_@MWCNT	64.3	35.7	14.9	19.5

**Table 3:** Production of FA by Mn_9_Mo_1_O_X_@MWCNT under different substrate conditions.

	Reaction Conditions							
Entry	Substrate	Qlty (g)	Cat (g)	H_2_O (mL)	*T* (°C)	Time (h)	*p* (O_2_) (Mpa)	FA Yield (%)
1	Fructose	0.1	0.05	5	140	6	3	49.9
2	Arabinose	0.1	0.05	5	140	6	3	56.8
3	Xylose	0.1	0.05	5	140	6	3	57.3
4	Cellobiose	0.1	0.05	5	140	6	3	34.4
5	Cellobiose	0.1	0.05	5	160	4	3	52.3
6	Xylan	0.1	0.05	5	140	6	3	50.7
7	Cellulose *^a^*	0.1	0.05	5	170	6	3	20.5
8	Cellulose *^a^*	0.1	0.05	5	180	4	3	23.5

*^a^* Cellulose pre-treated with ball milling and acid.

**Table 4:** Recent studies of glucose to FA with different metal oxides.

Catalyst	Concentration (g/L)	Cat (g)	*p* (O_2_)(Mpa)	*T* (°C)	Time (h)	Yield (%)	Ref.
Mn_9_Mo_1_O_X_@MWCNT	20	0.05	O_2_ (3)	140	6	58.8	♣
Mn_9_Mo_1_O_X_@MWCNT	60	0.05	O_2_ (3)	140	6	54.3	♣
Mn_9_Mo_1_O_X_@MWCNT	20	0.05	O_2_ (3)	130	10	54.4	♣
Mo(1)-MnO_X_	20	0.05	O_2_ (3)	160	1.5	65.8	[38]
MnO_X_-100	20	0.05	O_2_ (3)	160	2.5	67.2	[57]
VO_X_/TiO_2_	20	0.20	O_2_ (3)	150	2.5	42.0	[68]
H_8_[PV_5_Mo_7_O_40_]	18	0.16	O_2_ (2)	90	24	55.0	[69]
VOSO_4_	9	0.01	O_2_ (2)	140	1	45.0	[24]
Bi_1_V_2_O_X_	8.4	0.12	O_2_ (1)	170	0.34	62.0	[70]
HPA-5	22.5	1.74	O_2_ (6)	80	8	58.3	[71]
H_5_PV_2_Mo_10_O_40_	1 wt%	5 mol %	O_2_ (2)	100	3	52.0	[22]

♣ This work.

**Table 5:** The glucose conversion and FA yield over Mn_9_Mo_1_O_X_@MWCNT after three cycles.

Recycle Times	Catalyst Mass (mg)	Glucose Conversion (%)	FA Yield (%)
1	50 (fresh)	91.8	46.8
2	50 (used)	77.4	34.2
3	50 (used)	60.7	21.5

Condition: 100 mg glucose, 5 mL H_2_O, 3 MPa O_2_, 130 °C, 6 h.

## Data Availability

The data presented in this study are available on request from the corresponding author.
